# Cognitive and Social Cognitive Impairment and their Impact on Real-Life Functioning and Quality of life

**DOI:** 10.1192/j.eurpsy.2022.161

**Published:** 2022-09-01

**Authors:** G. Sachs

**Affiliations:** Medical University of Vienna, Psychiatry And Psychotherapy, Vienna, Austria

**Keywords:** Quality of Life, cognition, functional outcome, schizophrénia

## Abstract

Cognitive and social cognitive impairments are a central feature of schizophrenia and are known to significantly affect real-life functioning [1]. These impairments include deficits in memory, language function and executive function, as well as in processing speed and attention. In the domains of social cognition, face perception [2], voice perception, mentalizing and emotion regulation have been described to be affected. All deficits, cognitive and social-cognitive, can persist during symptomatic remission. Social cognition is a partial mediator between neurocognition and functional outcome. Recent research has demonstrated that neurocognition affects functional capacity and that social cognition affects community functioning [3]. The impact of cognition on quality of life (QOL) was shown in a large meta-analytic study, in which a moderate correlation of verbal ability and processing speed with subjective quality of life was found [4]. A network analysis showed that functional capacity and everyday life skills were the most central and highly interconnected nodes in the network. Functional capacity bridged cognition with everyday life skills, the everyday life skills node was linked to disorganization and expressive deficits [5]. Deficits in neurocognition and social cognition play a pivotal role as enduring impairment after clinical remission and as a critical rate-limiting factor in functional recovery. [1] Green et al. Schizophr Bull. 2000; 26(1): 119-136 [2] Sachs et al. Schizophr Res. 2004; 68(1):27-35 [3] Bechi et al. Psychiatry Res. 2017; 251:118-124 [4] Tolman & Kurtz Schizophr Bull. 2012; 272:419-424 [5] Galderisi et al. JAMA Psychiatry. 2018; 75(4):396-404

**Disclosure:**

No significant relationships.

